# Antipsychotic-like activity of Noni (*Morinda citrifolia* Linn.) in mice

**DOI:** 10.1186/1472-6882-12-186

**Published:** 2012-10-19

**Authors:** Vijayapandi Pandy, Megala Narasingam, Zahurin Mohamed

**Affiliations:** 1 Department of Pharmacology, Faculty of Medicine, University of Malaya, 50603 Kuala Lumpur, Malaysia

**Keywords:** Noni fruits, Apomorphine, Methamphetamine, Dopamine, Stereotypy, Cage climbing

## Abstract

**Background:**

Noni fruit is widely consumed in tropical regions of Indonesia to the Hawaiian Islands. The noni plant has a long history of use as a medicinal plant to treat a wide variety of ailments including CNS disorders. The present investigation was designed to evaluate the antipsychotic effect of noni fruits (*Morinda citrifolia* Linn.*)* using mouse models of apomorphine-induced climbing behaviour and methamphetamine-induced stereotypy (licking, biting, gnawing and sniffing).

**Methods:**

In acute study, the methanolic extract of *Morinda citrifolia* (MMC) at different doses 1, 3, 5, 10 g/kg was administered orally one hour prior to apomorphine (5 mg/kg, i.p) and methamphetamine ( 5 mg/kg, i.p) injection respectively in Swiss albino mice. In chronic studies, (TAHITIAN NONI® Juice, TNJ) was made available freely in daily drinking water at 30, 50 and 100% v/v for 7 days; 30 and 50% v/v for 21 days respectively. On the test day, an equivalent average daily divided dose of TNJ was administered by oral gavage one hour prior to apomorphine treatment. Immediately after apomorphine/ methamphetamine administration, the animals were placed in the cylindrical metal cages and observed for climbing behaviour/ stereotypy and climbing time.

**Results:**

The acute treatment of MMC (1, 3, 5, 10 g/kg, p.o) significantly decreased the apomorphine-induced cage climbing behaviour and climbing time in mice in a dose dependent manner. The MMC also significantly inhibited methamphetamine-induced stereotypy behaviour and climbing time in mice dose-dependently. The 7 and 21 days treatment of TNJ in drinking water at 50 and 100%v/v significantly alleviated the apomorphine-induced climbing behaviour and climbing time in mice.

**Conclusions:**

The present study results demonstrated the antidopaminergic effect of *Morinda citrifolia* Linn. in mice, suggesting that noni has antipsychotic-like activity which can be utilized in the treatment of psychiatric disorders. However further studies are warranted to identify the active principles responsible for the antipsychotic activity of noni.

## Background

Psychosis is a chronic recurrent neuropsychiatric disorder that alters the quality of life of the sufferers and it has been a major public health concern [1]. Current drug treatments are limited by poor efficacy and tolerability. Since psychiatric disorders are on the rise, clinicians are looking for alternative remedies and herbal medications for the treatment of neurobehavioral disorders. Noni is the general name given for the species *Morinda citrifolia* Linn (Rubiaceae) and is also known in different communities as Indian Mulberry, nunaakai (in Tamil), dog dumpling (Barbados), mengkudu (Indonesia and Malaysia), apatot (Philippines), kumudu (Bali), pace (Java), beach mulberry, cheese fruit or noni (Hawaii). Noni is an evergreen tree found growing in open coastal regions at sea level and in forest areas up to about 1300 feet above sea level. Noni is identifiable by its straight trunk, large, bright green and elliptical leaves, white tubular flowers and its distinctive, ovoid, “grenade-like” yellow fruit. The fruit can grow in size up to 12 cm or more and has a lumpy surface covered by polygonal-shaped sections. The seeds, which are triangular shaped and reddish brown, have an air sac attached at one end, which makes the seeds buoyant. The mature noni fruit has a foul taste and odour [2]. Noni juice has been used by the people of the South Pacific Islands for the past 2,000 years to aid a wide range of health illness. The fruit juice is in high demand as alternative medicine for different kinds of ailments such as arthritis, diabetes, high blood pressure, muscle aches and pains, menstrual difficulties, headaches, heart disease, AIDS, cancers, gastric ulcers, sprains, mental depression, senility, poor digestion, atherosclerosis, blood vessel problems, and drug addiction [3,4]. A wide variety of pharmacological activities have been reported for fruit, leaf and root extracts of noni such as analgesic [5], anti-inflammatory [6], antioxidant [7,8], immunomodulatory [9], anti-tumor [10], hepatoprotective [11], blood pressure lowering and vasodilatory [12,13], cardio protective [14], antifungal [15], phytoestrogenic [16], wound healing [17], insulinotrophic [18] and anti-osteoporotic activity [19]. Nevertheless, there have been only a few reports on the use of noni for CNS disorders such as anxiolytic and sedative [20], nootropic [21], antiepileptic [22], neuroprotective effect against stress-induced cognitive impairment [23] and some neuropharmacological effects [24].

*Morinda citrifolia* is regarded as a safe antiemetic drug in primary health care in Thailand [25]. The decoction or infusion of roasted mature unripe noni fruits is recommended to relieve the symptoms of mild nausea and vomiting [26]. The prokinetic and antiemetic actions of noni fruit extract were detected by Chuthaputti et al. [27] in which they found that the intestinal transit was delayed by apomorphine (a potent agonist of dopamine D_2_ receptor) in mice meanwhile emesis induced by apomorphine was reduced in dogs. The results indicated that an aqueous extract of noni fruit at the dose equivalent to crude drug 10–20 g/kg body weight and also at the dose equivalent to crude drug 40 g/kg body weight, might contain a weak antidopaminergic agent responsible for its prokinetic in mice and an antiemetic effect observed in humans respectively [25]. Conversely, it was reported that the administration of *Morinda citrifolia* fruit extract significantly increased the brain levels of monoamines including dopamine in rats [22]. These contradictory results led us to conduct further studies of noni on the dopaminergic system. Hence the present study is designed to evaluate the neuromodulatory effect of noni (*Morinda citrifolia* Linn*)* on the dopaminergic system using firstly, mouse model of apomorphine-induced climbing behaviour and climbing time and secondly, methamphetamine-induced stereotypy (licking, biting, gnawing and sniffing) and climbing time.

## Methods

### Plant material

Fresh unripe fruits (dark green) of *M. citrifolia* were collected on January 2012 from Malacca, Malaysia. The plant material was taxonomically identified and authenticated by Rimba Ilmu, Institute of Biological Sciences, University of Malaya and the voucher specimen (KLU 47738) was deposited at Rimba Ilmu for future reference. The fruits were cut into thin slices and shade dried. The shade dried fruit slices were pulverized in a mechanical grinder to obtain a coarse powder.

### Preparation of *Morinda citrifolia* fruit extract

The methanolic extract of *Morinda citrifolia* (MMC) was prepared using cold extraction with sonication. The coarse fruit powder (1.8 kg) was soaked with 10 L of methanol (Scharlau, Spain, isocratic HPLC grade) for 20 h followed by sonication using a water bath sonicator at 30°C for another 4 h. The resultant solution was evaporated under vacuum in a rotary evaporator to obtain a dry mass extract (yield: 14.36% w/w). The dried solvent free crude MMC was stored in 4°C in an airtight, labelled, amber-colored container until further use.

### Animals

Swiss albino male mice (25-30 g), obtained from the laboratory animal centre, University of Malaya were used in this study. The mice were housed in polycarbonate cages in a group of six to seven animals under standard laboratory conditions at temperature of 22±1°C and 12 h light: 12 h dark cycle. Animals were fed with standard laboratory food pellet and water *ad libitum*. The animals were acclimatized to the experimental room and handled for one week prior to start of the experimentation. Animal Care and Use Committee, Faculty of Medicine, University of Malaya, Kuala Lumpur approved the experimental protocol (ACUC Ethics No. FAR/27/01/2012/PV (R)) and care of the animals were taken as per guidelines of the Council for International Organization of Medical Sciences (CIOMS) on animal experimentation [28].

### Drugs and chemicals

Apomorphine hydrochloride and sodium metabisulphite (Sigma-Aldrich, USA), haloperidol (Manace Injection®, Duopharma (M) SDN BHD, Malaysia) and methamphetamine hydrochloride (MOSTI, Malaysia) were used. All the drug solutions were prepared fresh in normal saline and administered intraperitoneally (i.p) in a constant volume of 1 ml/100 g body weight of the animal. Apomorphine hydrochloride was dissolved in saline containing sodium metabisulphite (0.125% w/v). The MMC was suspended in 1% w/v sodium carboxy methyl cellulose (CMC) solution and administered orally (p.o). CMC solution was served as vehicle control (VEH). Commercial noni fruit juice, TNJ was obtained from Morinda International Inc, Malaysia. TNJ is a fruit mixture of 89% noni fruit puree and 11% of grape and blueberry juice concentrate and natural flavours because 100% pure noni juice is unpalatable.

### Statistical analysis

The data are expressed as mean ± S.E.M. The statistical significance of differences between groups were evaluated by one way analysis of variance (ANOVA) followed by the student's t-test. Stereotypy and climbing behaviour was analysed by Kruskal-Wallis test followed by Mann–Whitney *U*-test because nonparametric statistics are needed with all or none or rating scale scores. All data analyses were conducted using GraphPad Prism 5 statistical software. A level of p < 0.05 was considered statistically significant.

### Experimental design

#### Apomorphine-induced climbing behaviour in mice

Administration of apomorphine to mice results in a peculiar climbing behaviour characterized initially by rearing and then spontaneous climbing activity [29]. A cylindrical metal cage (18×19 cm) consisting of vertical (1 cm apart) and horizontal (4.5 cm apart) metal bars (2 mm) with upper lid was used in the present study. In acute studies, test groups received different doses of MMC (1, 3, 5 and 10 g/kg, p.o), and the vehicle-treated group received 1% w/v CMC solution (1 ml/100 g, p.o), one hour before apomorphine injection (5 mg/kg, i.p). The reference drug, haloperidol (dopamine D_2_ receptor antagonist) treated positive control group received a dose (2 mg/kg, i.p) 30 min before apomorphine injection. In chronic studies, TNJ was made available freely in the daily drinking water at 30, 50 and 100% v/v for 7 days; 30 and 50% v/v for 21 days respectively in different sets of animals. The mean daily dose of noni juice based on daily intake of water was calculated and found to be ~6, 10 and 13 ml/100 g/d for 30, 50 and 100% v/v respectively. On the test day, an equivalent average daily divided dose of TNJ was administered by oral gavage one hour prior to apomorphine treatment. Initially, naïve mice were placed individually at the base of the cage for 15 min to explore freely. Immediately after apomorphine administration, the animals were placed back into the corresponding metal cages and observed for climbing behaviour. An observer who was blind to drug treatment measured the total time spent on the wall of the cage whereby the climbing behaviour of individual mice was scored at 5-min intervals for a period of 30 min. The scoring system used as follows: 0 = four paws on the floor, 2 = two paws on the wall of the cage, 4 = four paws on the wall of the cage (climbing) and the score corresponding to the posture the animal adopted the longest were recorded. Climbing scores across each 5 min interval for a period of 30 min were then summarized and expressed as climbing index, thus providing a maximum possible climbing index of 24.

#### Methamphetamine-induced stereotypy and climbing time in mice

The apparatus and procedures that were used are the same as described elsewhere [30] with some modifications. They were randomly assigned to each drug regimen and received the MMC (1, 3 and 5 g/kg, p.o) one hour before methamphetamine (5 mg/kg, i.p) injection. The positive control group received haloperidol (2 mg/kg, i.p) 30 min before methamphetamine injection. Naïve mice were initially placed individually in cylindrical metal cages described in the previous experiment for 15 min to acclimatize to the new environment. Immediately after methamphetamine administration, the mice were placed inside the cage at its base. After 30 min of methamphetamine administration, the animal was placed on the inner lid of the cage and lid was closed. Methamphetamine does not induce spontaneous climbing behaviour as apomorphine does and hence when the methamphetamine treated animal is placed on the lid of the cage, it remained on it for a longer time while the saline treated animal will immediately go down to the base. The total time spent on the inner lid/ wall of the cage was measured for 30 min. The intensity of stereotyped behaviour of individual mice was scored at 15-min intervals for a period of 60 min. The scoring system used was: Score 0 (no change compared to control), 1 (discontinuous sniffing, constant exploratory activity), 2 (continuous sniffing, periodic exploratory activity), 3 (continuous sniffing, discontinuous biting, gnawing or licking), and 4 (continuous biting, gnawing or licking; no exploratory activity).

#### Safety study

Animals were divided in groups of six each. The acute oral toxicity study was performed in test groups that were treated with either MMC (20 g/kg), or TNJ (20 ml/100 g, 4 divided doses in 30 min interval). The maximum volumes of administration in mice used to be 5 ml/100g. Vehicle treated group received 1%w/v CMC (1 ml/100 g, p.o) served as the control. The mice were allowed food and water *ad libitum* during the 24 h test period and kept under regular observation of gross physiological, behavioural changes (skin state, salivation, whimpering, trembling, locomotion and excretion) and mortality. Body weight of each mouse was recorded daily [5,13].

## Results

### Apomorphine-induced climbing behaviour in mice

**Figure 1:**
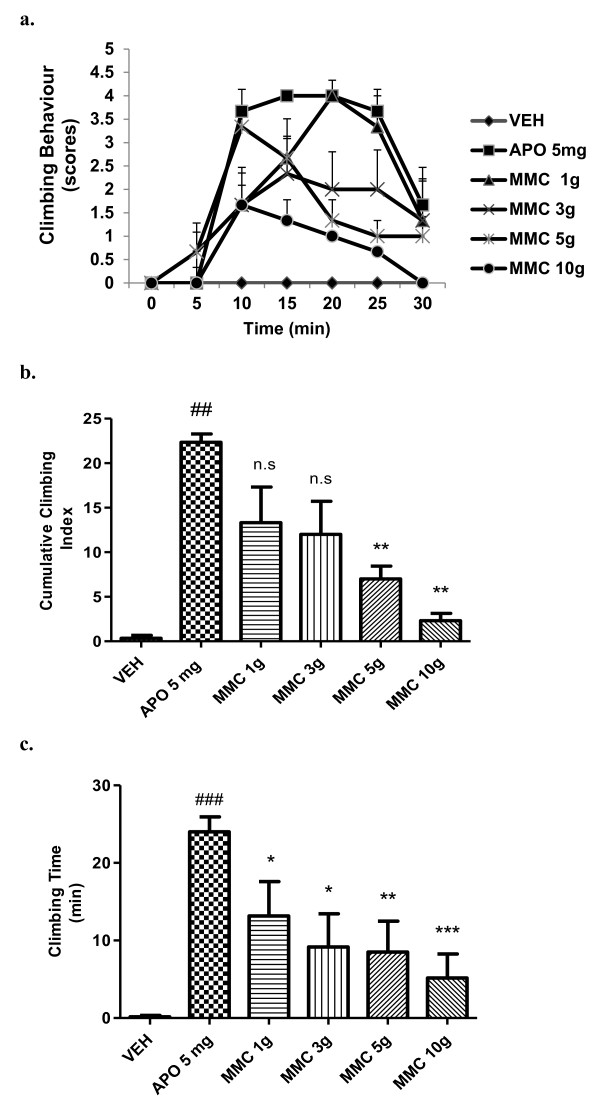
**Effect of MMC, (g/kg, p.o) on cage climbing behaviour and climbing time induced by apomorphine in mice.** (**a**) Each point represents the mean ± SEM from the scores obtained from six animals. (**b**) The cumulative scores were measured for 30 min after administration of apomorphine. (**c**) Total time spent on the wall of the cage. ### *p* < 0.001, ## *p* < 0.01 compared with that of the saline group; * *p<* 0.05, ** *p* < 0.01, ***p <0.001 compared with that of the apomorphine (APO, mg/kg, i.p) group. Haloperidol (2 mg/kg, i.p) showed 100% inhibition of apomorphine response (data not shown). n.s- not significant.

Pre-treatment with the MMC (1, 3, 5 and 10 g/kg, p.o) significantly (p< 0.01) inhibited apomorphine-induced climbing behaviour (Figure 1a and b). Similarly, MMC (1, 3, 5 and 10 g/kg, p.o) significantly [*F (*5*,* 30*)* = 5.825; *P <*0*.*01] reduced the total time spent on the wall of the cage in apomorphine-induced mice dose-dependently (Figure 1c). MMC (1- 10 g/kg, p.o) *per se* did not produce either climbing behaviour or ataxia in this experiment, when compared to the vehicle control group (data not shown).

**Figure 2:**
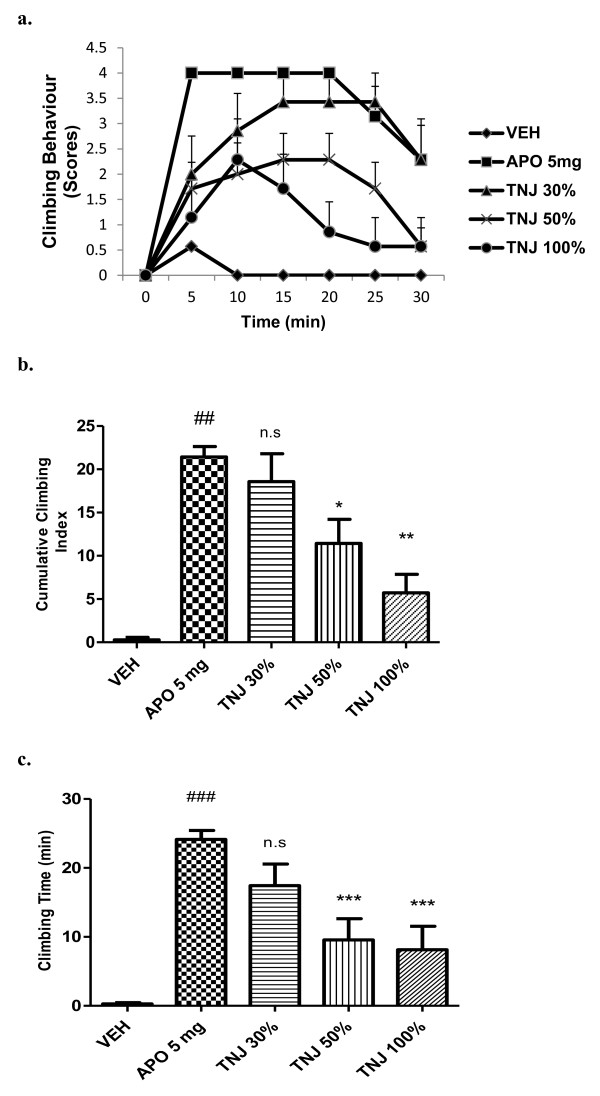
**Effect of TNJ, (% v/v, 7 days in the drinking water) on cage climbing behaviour and climbing time induced by apomorphine in mice.** (**a**) Each point represents the mean ± SEM from the scores obtained from seven animals. (**b**) The cumulative scores were measured for 30 min after administration of apomorphine. (**c**) Total time spent on the wall of the cage. ### *p* < 0.001, ## *p* < 0.01 compared with that of the saline group; * *p<* 0.05, ** *p* < 0.01, ***p <0.001 compared with that of the apomorphine (APO, mg/kg, i.p) group. n.s- not significant.

The 7 days pre-treatment with TNJ in drinking water at 30, 50 and 100 %v/v significantly (p< 0.01) alleviated apomorphine-induced climbing behaviour (Figure 2a and b). TNJ also significantly [*F (*4*,* 30*)* = 12.88; *P <*0*.*01] declined the total time spent on the wall of the cage in apomorphine-induced mice dose-dependently (Figure 2c).

**Figure 3:**
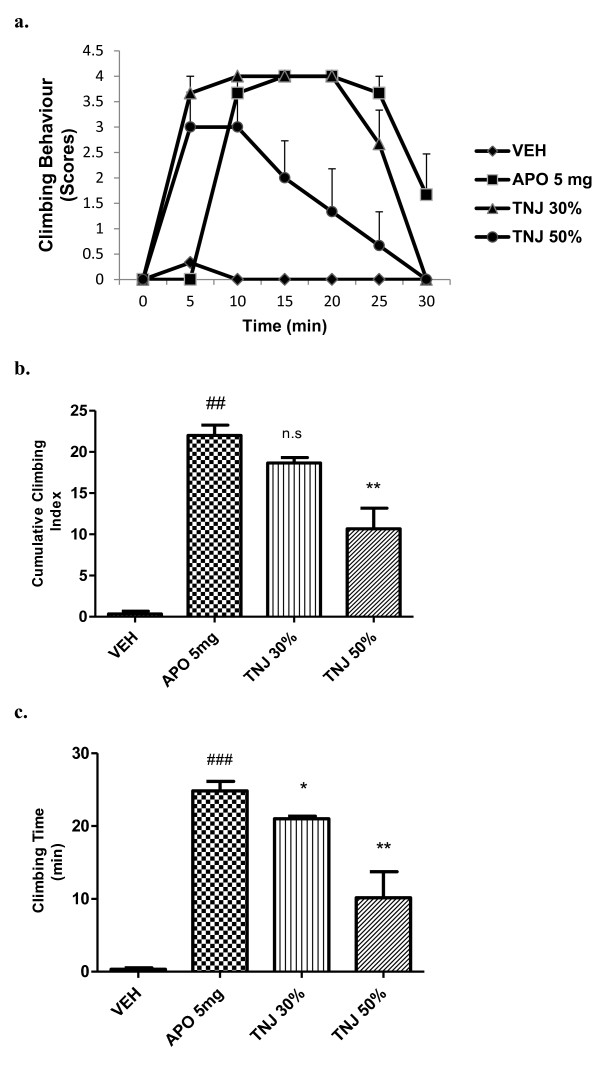
**Effect of TNJ, (% v/v, 21 days in the drinking water) on cage climbing behaviour and climbing time induced by apomorphine in mice.** (**a**) Each point represents the mean ± SEM from the scores obtained from six animals. (**b**) The cumulative scores were measured for 30 min after administration of apomorphine. (**c**) Total time spent on the wall of the cage. ### *p* < 0.001, ## *p* < 0.01 compared with that of the saline group; * *p<* 0.05, ** *p* < 0.01, ***p <0.001 compared with that of the apomorphine (APO, mg/kg, i.p) group . n.s- not significant.

Pre-treatment with TNJ (30 and 50%v/v) for 21 days also significantly [*F (*3*,* 20*)* = 33.36; *P <*0*.*01] reduced climbing behaviour and total time spent on the wall of the cage in apomorphine-induced mice (Figure 3a,b and c). Haloperidol (2 mg/kg, i.p) completely reversed the apomorphine-induced climbing behaviour and climbing time in mice (data not shown).

### Methamphetamine-induced stereotypy and climbing time in mice

**Figure 4:**
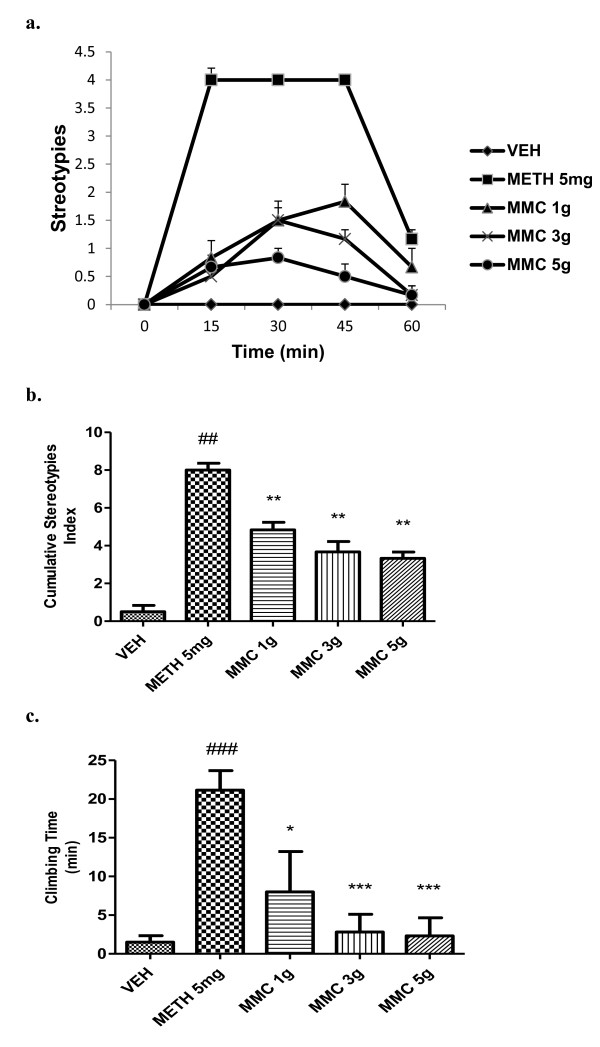
**Effect of MMC, (g/kg, p.o) on stereotypies and climbing time induced by methamphetamine in mice.** (**a**) Each point represents the mean ± SEM from the stereotypy scores obtained from six animals. (**b**) The cumulative stereotypy scores were measured during 30 to 60 min after administration of methamphetamine. (**c**) Total time spent on the wall of the cage. ### *p* < 0.001, ## *p* < 0.01 compared with that of the saline group; *p< 0.05, ** *p* < 0.01, ***p <0.001 compared with that of the methamphetamine (METH, mg/kg, i.p) group. Haloperidol (2 mg/kg, i.p) showed 100% inhibition on methamphetamine-induced stereotypies in mice (data not shown). n.s- not significant.

Pre-treatment with MMC (1, 3, 5 g/kg, p.o) significantly (p<0.0001) inhibited methamphetamine-induced stereotypy behaviour in a dose-dependent manner (Figure 4a and b). It also significantly [*F (*4*,* 25*)* = 7.535; *P <* 0*.*01] attenuated methamphetamine-induced climbing time in mice (Figure 4c). Haloperidol (2 mg/kg, i.p) completely reversed the methamphetamine-induced stereotypy and climbing time in mice (data not shown).

### Safety study

The acute oral treatment with MMC at 20 g/kg and TNJ at 20 ml/100 g did not produce any mortality or changes in gross behaviour, as compared to vehicle treated group when observed for 14 days.

## Discussion

The behavioural responses observed in animals after administration of the dopamine agonist, apomorphine are attributed to activation of D_1_ and D_2_ receptors [31,32]. Mesolimbic and nigrostriatal dopaminergic pathways play key roles in the mediation of locomotor activity and stereotyped behaviour. Stereotyped behaviour is more closely associated with the caudate striatum area of the brain [31-33]. Animal models used for screening antipsychotic drugs are based on the neurochemical hypothesis of schizophrenia, involving mainly the neurotransmitters dopamine and glutamate [34]. Antagonism of dopamine D_2_ receptors may be a common feature of most clinically effective antipsychotic drugs, especially those active against hallucinations and delusions [35]. The dopamine-based models usually employ apomorphine, a direct agonist, or amphetamine, a drug that increases the release of this neurotransmitter and blocks its re-uptake. In the present study, acute pre-treatment of MMC, (1-10 g/kg, p.o) in mice showed a significant dose-dependent decrease in climbing behaviour and climbing time induced by apomorphine. In addition, 7 and 21 days pre-treatment with TNJ (30, 50 and 100% v/v in daily drinking water) also significantly alleviated apomorphine-induced climbing behaviour and climbing time in mice. The reference drug, haloperidol (2 mg/kg, i.p) completely reversed the apomorphine-induced climbing behaviour and climbing time in mice. Margarita et al [24] demonstrated neuroleptic activity of the juice of the ripe fruit of the noni in mice. Noni juice at different doses (5, 10 and 100 ml/kg, i.p) equivalent to dried juice powder doses (450, 900 and 1800 mg/kg, i.p) significantly reduced stereotypies induced by amphetamine (3 mg/kg, s.c) in a dose-dependent manner [24]. The present study results are consistent with these findings and revealed MMC and TNJ might have dopamine D1 and/or D2 receptors antagonistic phytoconstituents.

To further confirm these findings, another set of experiment was carried out by replacing apomorphine with methamphetamine. Methamphetamine is a member of the family of phenethylamine. Methamphetamine causes the norepinephrine, dopamine, and serotonin (5HT) transporters to reverse their direction of flow. This inversion leads to a release of these transmitters from the vesicles to the cytoplasm and from the cytoplasm to the synapse, causing increased stimulation of post-synaptic receptors. Methamphetamine also indirectly prevents the reuptake of these neurotransmitters, causing them to remain in the synaptic cleft for a prolonged period [36]. It would then produce an effect that is similar to the apomorphine-induced behaviour in mice which in this case is stereotypy. Stereotyped licking, biting, and other orofacial behaviours are known to involve nigrostriatal dopaminergic neurotransmission [37] in distinct striatal sub regions [38-40]. Importantly, it appears that control of sniffing and biting is mediated by different striatal sub regions [38]. Overall, mesolimbic dopaminergic mechanisms have been proposed to play a critical role in the expression of stereotypy after acute psychostimulant administration [41]. Since some of the specific behaviours produced in these models closely resemble that of humans abusing amphetamines, animals that display stereotypy have been considered as an animal model for amphetamine psychosis and are considered to be particularly relevant to schizophrenia [42]. However, because of the compulsive and the repetitive nature of the behaviour, amphetamine-induced stereotypy have also been considered as potential animal models of obsessive–compulsive disorder [43] and autism [44]. Methamphetamine-induced stereotypy persists for several hours in rodents, and this abnormal behaviour can be reversed by dopamine antagonists [45], but less so by other agents [46]. The acute pre-treatment of MMC (1, 3 and 5 g/kg, p.o) significantly decreased the methamphetamine-induced stereotypy and cage climbing time in a dose-dependent manner. These results further confirm the antidopaminergic effect of MMC of noni unripe fruits.

Analgesic properties for commercial noni juice in rats have been reported in the literature. The results showed that rats fed with 10% and 20% noni juice had greater pain tolerance (162% and 212%, respectively) compared with the placebo group [3]. Noni root extract (1600 mg/kg) showed significant analgesic activity in mice through the writhing and hotplate tests, similar to the effect of morphine (75% and 81% protection using noni extract and morphine, respectively), and it was also proven to be non-toxic [5,47]. These studies suggested that the central pharmacological effects of noni can be observed at higher doses [24]. In the present study, the antidopaminergic effects of MMC and TNJ were observed only at higher doses.

Several oral toxicity studies in Sprague-Dawley rats, using the widely consumed commercial noni fruit juice, TNJ have been assessed [3,48]. Acute and sub chronic (13 wks.) oral toxicity studies revealed no diverse effects from consuming doses equivalent to 80 ml/kg body weight/d [48]. Pureed noni fruit from Tahiti was administered by oral gavage at a dose of 15 g/kg to Sprague-Dawley rats. All animals survived and showed no signs of toxicity or behavioural changes when observed for two weeks. Conversely, all animals appeared healthy and gained weight. Gross necropsies of all animals at the end of 2 weeks revealed no pathological effects. Consequently, the LD50 of noni fruit was found to be greater than 15 g/kg [49]. Compounds are considered nontoxic if the acute oral LD50 is greater than 5 g/kg, or if the acute intraperitoneal LD50 is greater than 2 g/kg. The LD50 of noni fruit juice and its crude extract are greater than the minimum criteria for nontoxic status [48]. The present acute toxicity results are consistent with these earlier reports. In the present study, acute oral treatment of TNJ at 200 ml/kg and MMC at 20 g/kg respectively did not show any toxic effects and behavioural changes when observed for 14 days.

It has been demonstrated that *M. citrifolia* possess antiemetic property in patients who are considered high risk for postoperative nausea and vomiting (PONV) after various types of surgery. The 600 mg dose of noni extract (equivalent to 20 g of dried noni fruit/ 8.712 mcg of scopoletin) was the minimum dose that effectively reduced the incidence of postoperative nausea in the early postoperative period [50]. However, this study could not reveal the possible mechanism of action of *M. citrifolia* for antiemetic action. Our study result suggests that the antiemetic activity of *M. citrifolia* might be mediated through dopaminergic pathways. It is well known that dopamine D_2_ receptors in the area postrema play an important role in the regulation of emetic responses in ferrets, dogs and humans [51,52]. From a clinical point of view, dopamine receptor antagonists such as phenothiazines, butyrophenones and benzamides, which have affinity for dopamine D_2_ and D_3_ receptors, are used as antiemetic agents [53]. The contributions of dopaminergic abnormalities to the pathophysiology of schizophrenia have been studied most intensively. The focus on dopaminergic abnormalities in schizophrenia was prompted by the complementary observations in humans that psychosis can be elicited by psychostimulant medications such as d-amphetamine, especially when they are abused, whereas the ability to inhibit competitively the binding of dopamine to the D_2_ type of dopamine receptor is a pharmacological property shared by all of the conventional antipsychotic medications [54]. The obtained antidopaminergic activity of *M. citrifolia* in the present studies could be utilized in the treatment of schizophrenia.

Conversely, it has been demonstrated that ethyl acetate fraction of crude methanol extract of *Morinda citrifolia* at a daily dose of 200 and 400 mg/kg when administered to rats for 15 days significantly increased the brain levels of serotonin, dopamine and noradrenaline, which could be attributed to the significant protection offered against MES induced seizures in rats [22]. These opposing effects could be due to dose differences. This kind of biphasic response was extensively studied and reported in the literature [55]. However, further neurochemical studies in the brain are necessary to confirm this hypothesis and such studies are indeed underway in our laboratory.

Phytochemical studies using high performance liquid chromatographic (HPLC) fingerprint profile of the MeOH extracts of noni fruit revealed three major peaks representing scopoletin, rutin and quercetin (retention times: 25.72, 28.57 and 32.70 min, respectively), together with several minor peaks. These are major bioactive constituents of noni responsible for various pharmacological activities [20,56]. The antipsychotic like-effect of MMC and TNJ observed in the present study might be attributed to the presence of these phytoconstituents. Investigation of antidopaminergic active phytoconstituents responsible for noni's antipsychotic effect by using bioassay-guided chromatographic fractionation is currently underway in our laboratory.

## Conclusions

In conclusion, this study provides evidence that MMC dose-dependently attenuated the stereotyped behaviour induced by apomorphine and methamphetamine respectively in Swiss albino mice. In addition, the commercial noni juice TNJ when administered in drinking water for 7 and 21 days respectively also alleviated apomorphine-induced stereotypy in mice. These observed effects might be attributed to dopaminergic antagonistic and/or the reduction of dopamine availability in the brain. The antidopaminergic activity of noni fruits might be responsible for the traditional claim of effective treatment of nausea and vomiting. However, further studies are warranted to isolate and characterize these compounds for the effective utilization in the treatment of various kinds of emesis and psychiatric disorders.

## Competing interests

The authors declare that they have no competing interests.

## Authors’ contributions

PV designed the study, participated in the experiments and drafted the manuscript. MN performed the experiments, acquired the data, and accomplished the data analysis. ZM participated in study design and critically revised the manuscript for important intellectual content. All authors read and approved the final manuscript.

## Pre-publication history

The pre-publication history for this paper can be accessed here:

http://www.biomedcentral.com/1472-6882/12/186/prepub
